# Postinfektiöse Luxation der M.‑tibialis-posterior-Sehne einer Primaballerina: Ein 13-Jahres-Fallbericht

**DOI:** 10.1007/s00113-025-01597-4

**Published:** 2025-06-30

**Authors:** Hans Zwipp

**Affiliations:** UniversitätsCentrum für Orthopädie, Unfall- und Plastische Chirurgie, Universitätsklinikum Carl Gustav Carus der TU Dresden, Fetscherstraße 74, 01307 Dresden, Deutschland

**Keywords:** Ersatz des osteofibrösen M.-tibialis-posterior-Sehnen-Köchers, Pathologische Retinaculum-Ruptur, Klassifikation der M.-tibialis-posterior-Sehnen-Luxation, Lokale Kortikosteroidinjektion, Putride Sehneninfektion, Replacement of the osteofibrous tibialis posterior tendon quiver, Pathological retinaculum rupture, Classification of posterior tibialis tendon dislocation, Local corticosteroid injection, Putrid tendon infection

## Abstract

**Hintergrund:**

Lokale Kortikosteroidinjektionen induzieren bei Sportlern als häufigste Nebenwirkung Rupturen kollagenen Gewebes wie Faszien und Sehnen.

**Ziel der Arbeit:**

1. Warnung vor Cortisoninjektionen in der Nähe von Sehnen, Faszien und Retinacula. 2. Vorstellung der anatomischen Wiederherstellung des osteofibrösen Köchers der M.-tibialis-posterior-Sehne. 3. Einführung einer pathogenetischen Klassifikation der M.-tibialis-posterior-Sehnen-Luxation.

**Material und Methode:**

Eine 23-jährige Balletttänzerin litt 8 Monate nach radikalem Débridement wegen putrider Infektion mit *Klebsiella pneumoniae* der linken M.-tibialis-posterior-Sehne (MTPS) bei sonographisch erkennbarer Luxation derselben trotz straffen Tapings an Schmerzen und signifikantem Kraftverlust beim Tanz en pointe. Nach Débridement der teilrupturierten, stark aufgespleißten Sehne erfolgte nach Reposition derselben eine anatomische Wiederherstellung des osteofibrösen Sehnenköchers mit ortsständigem Gewebe. Perioperative Antibiotika, ein postoperativer Unterschenkel-Geh-Cast für 6 Wochen mit Thromboseprophylaxe und intensive Rehamaßnahmen führten nach 8 Wochen zur vollständigen Arbeitsfähigkeit.

**Ergebnis:**

Im 13-jährigen Verlauf erfolgte kein Rezidiv. Bei schmerzfreiem Tanzen en pointe ohne Taping ist die heute 36-jährige Solistin unverändert erste Tänzerin an einem namhaften deutschen Opernhaus, inzwischen Mutter zweier Kinder, und meint, heute besser zu tanzen als früher.

**Diskussion:**

Ob die initiale lokale Kortikosteroidinjektion eine pathologische Retinaculumruptur mit Luxation der MTPS bedingte oder ob sie ursächlich die putride Infektion nach einer Sehnenrevisonsoperation auslöste, bzw. ob ein nekrotisches Retinaculum dem radikalen Débridement anheimfiel, musste offen bleiben.

## Abstract (en)

**Background:**

The most frequent side effect of local corticosteroid injections in athletes is the rupture of collagenic tissues, such as fascia and tendons.

**Objective:**

1. Warning against cortisone injections in the vicinity of tendons, fascia and retinacula. 2. Presentation of the anatomical restoration of the osteofibrous quiver of the tibialis posterior tendon. 3. Introduction of a pathogenetic classification of tibialis posterior tendon dislocation.

**Material and method:**

A 23-year-old female ballet dancer suffered from pain and significant loss of strength despite stiff taping while dancing en pointe 8 months after radical debridement for a putrid infection with *Klebsiella pneumoniae* of the left tibialis posterior tendon with a sonographically detectable dislocation. After debridement and reduction of the partially ruptured, scarred and split tendon, an anatomical restoration of the osteofibrous tendon quiver was performed with local tissue and transosseous sutures. The targeted perioperative administration of antibiotics, a postoperative lower leg cast for 6 weeks with thrombosis prophylaxis and subsequent intensive rehabilitation measures resulted in the complete ability to work after 8 weeks.

**Result:**

There were no recurrences over the 13-year period. With pain-free dancing en pointe without taping, the now 36-year-old soloist is still the lead female dancer at a renowned German opera house, is the mother of two children and believes that she dances better today than before.

**Discussion:**

Whether the initial local corticosteroid injection caused a pathological retinaculum rupture with dislocation of the tibialis posterior tendon or whether it was originally caused by the putrid infection after tendon revision surgery or whether a necrotic retinaculum was ultimately subjected to radical debridement, must remain an open question.

## Einleitung

Die isolierte Luxation der M.-tibialis-posterior-Sehne ist eine seltene Entität. Nach einer allerersten Fallbeschreibung im Jahr 1874 durch Charles Martins, Professor für Botanik und Medizin in Montpellier, der diese Entität selbst beim Ballonabsturz 1871 erlitt [23], erfassten Lohrer und Nauck [20] von 1874 bis 2007 mittels systematischem Review der internationalen Literatur nur 61 Fälle einer MTPS-Luxation. Von 2008 bis 2024 kamen weitere 18 Einzelfallberichte hinzu [1, 6, 9, 19, 24, 27, 34, 36, 39, 41, 47]. Obwohl seit den 1980er-Jahren Sonographie und MRT eine sichere Diagnose dieser Entität erlauben, analysierten Lohrer und Nauck [20] eine extrem hohe Fehldiagnose in 53,1 % der Fälle. Es erscheint daher wichtig, nach 150 Jahren über den vermutlich 80. Fall zu berichten, zumal eine postinfektiöse Genese bisher nicht vorgestellt und diskutiert wurde.

## Anatomie, Biomechanik und Pathophysiologie

Der M. tibialis posterior wird vom N. tibialis innerviert. Er entspringt auf Höhe der proximalen Membrana interossea cruris und geht handbreit oberhalb des Innenknöchels in seine extrinsische Sehne über, die in einem Sulcus hinter dem Innenknöchel innerhalb einer Vagina synovialis geführt und durch einen osteofibrösen Köcher als Teil des Retinaculum musculorum flexorum gesichert wird. Sie verläuft weiter distal medial-plantar unter den Taluskopf, den sie – unter dem Pfannenband plantarseitig verlaufend – aktiv stützt, um dann meist mit 5 Armen, davon mit kurzen Fasern an der Tuberositas ossis navicularis, mit langen Fasern am plantaren Anteil des Os cuneiforme mediale sowie mit 3 weiteren, dünneren Schenkeln plantarseitig am Os cuneiforme laterale und an den Basen der Ossa metatarsalia II–IV zu inserieren [44, 48]. Der M. tibialis posterior ist nach Lanz-Wachsmuth [17] biomechanisch einer der Flexoren, v. a. aber mit 1,5 Nm Arbeitsleistung nach dem M. triceps surae der kräftigste Supinator und der wichtigste Antagonist zu den Mm. peronaei. Seine Funktionen sind die Beendigung der Pronationsphase beim Bodenkontakt der Fußsohle zu Beginn des Abrollvorgangs sowie die Stabilisierung des Subtalar- und Chopart-Gelenks zur Erzeugung eines rigiden Hebelarms für den Abstoß des Fußes [17, 44, 49]. Diese essenzielle Funktionen kann diese Muskel-Sehnen-Einheit nur bei intaktem Retinaculum und dem ihr eigenen osteofibrösen Köcher erfüllen.

*Pathophysiologisch* geht die Dysfunktion der M.-tibialis-posterior-Sehne mit der Entwicklung eines Pes planovalgus einher, was nach Mikrorupturen bei OSG-Pronations-Abduktions-Frakturen [48] und nach Makroruptur der Sehne beim dekompensierten Pes plano valgus diverser Genese [49], aber auch nach übersehener traumatischer Luxation der Sehne beobachtbar ist [12, 49].

## Epidemiologie und Ursachen

Nach Al Khudairy et al. [1] waren in 39 Fällen ihrer Literaturrecherche 24 Männer und 15 Frauen im mittleren Alter von 31 (12 bis 56) Jahren betroffen. Ursächlich wurde 13-mal Sport, 11-mal Trauma, inkl. 2 Verkehrsunfälle, 11-mal keinerlei Trauma und 4‑mal eine andere Ursache wie Voroperationen oder langfristige Kortisoninjektionen (Fall von [31]) angegeben.

Lohrer und Nauck [20], die bis 2007 im Review in 36 internationalen Berichten insgesamt 61 Fälle analysierten, fanden in 58,5 % als Luxationsursache ein Trauma beim Sport. Während die meisten Betroffenen einen Fehltritt, ein Stolpern mit Umknicken des Fußes, meist im Sinne des Pronation-Eversion-Traumas unter gleichzeitiger Extension im OSG angaben, war in immerhin 15 % keinerlei Trauma eruierbar. Kontrovers zum Unfallhergang beschrieb zuvor 1969 Scheuba [37] im ersten deutschsprachigen Bericht, dass ein 33-jähriger Tennisspieler im Zehenspitzenstand bei ausführendem Rückschlag eine schmerzhafte Luxation der MTPS erlitt. Akute Luxationen bei Fehlen jedes erkennbaren Traumas beim Fußballspiel beschrieben andere Autoren [30, 42]. Ouzounian und Myerson [31] erklärten in ihrer eigenen Fallserie von 7 Fällen die Ursache 6‑mal durch Verdrehtraumen des Fußes und einmal durch lokale Kortisoninjektionen über 18 Monate.

Auch direkte Traumen beim Eishockey durch einen Puck-Treffer gegen den dorsalen Innenknöchel [15] oder einen Tritt durch den gegnerischen Spieler beim Fußball [26] wurden als ursächliche Auslöser beschrieben, im Puck-Treffer-Fall kompliziert durch Ausbildung eines Ganglions mit rezidivierender Luxation.

Selbst eine kombinierte MTPS-Luxation mit simultaner Achillessehnenruptur wurde berichtet [6].

Eine kürzlich erstellte CT-Analyse an 110 Personen ohne MTPS-Pathologie zu Weite und Tiefe des Sulcus tendinis musculi tibialis posterioris [46] zeigte, dass die Weite einer flach-konkaven Form geschlechtsspezifisch variiert, nicht aber die mittlere Tiefe von nur 1,6 mm in 80,2 %, was als Prädisposition zur Luxation der Sehne diskutiert wurde.

Bedenkt man, dass sich Luxationen der MTPS bei komplexen Brüchen des Pilon tibiale mit Inkarzeration der Sehne in 16 %, bei komplizierten trimalleolären OSG-Frakturen in 43 % [40], selbst vereinzelt bei isolierter Fibula- [33] oder Malleolus-medialis-Fraktur [49] bzw. bei der Luxatio pedis sub talo lateralis [49] begleitend ereignen, dürfte die Inzidenz insgesamt noch höher sein als bisher bekannt. Im vorliegenden 13-Jahres-Fallbericht wird erstmals ursächlich über eine mögliche postinfektiöse Luxation berichtet.

## Diagnostik

**Abb. 1:**
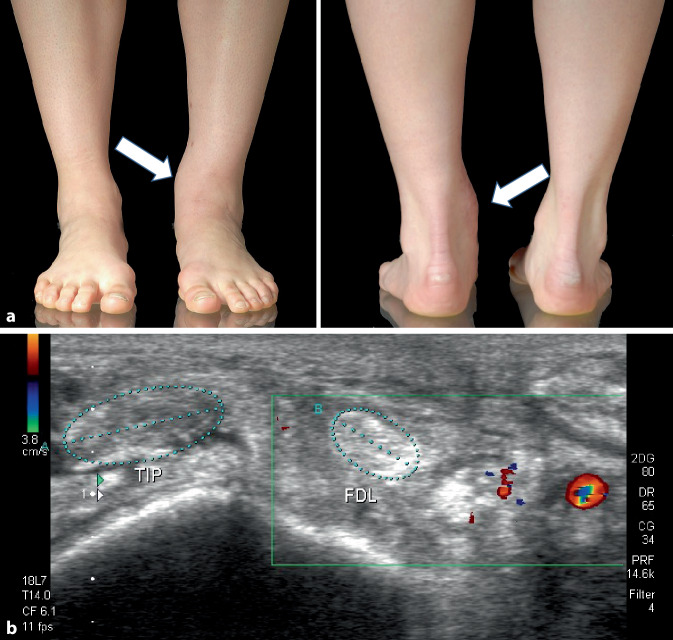
**a** Klinisch verstrichene Kontur des Innenknöchels (*Pfeile*) bei luxierter M.-tibialis-posterior-Sehne **b** Koronarer Sonographieschnitt auf Höhe der nach ventral breit ausgezogenen M.-tibialis-posterior-Sehne (*TIP*), in situ befindliche M.-flexor-digitorum-Sehne (*FDL*)

Bei der *klinischen *Untersuchung sind bei akuter Luxation Schwellung, Schmerz und Ekchymose im Innenknöchelbereich beobachtbar. Differenzialdiagnostisch sind eine ligamentäre oder ossäre Pronation-Eversion-Läsion wie die mediale Bandzerrung, die Deltoidruptur oder Innenknöchelfraktur abzugrenzen. Bei subakuten und chronischen Fällen ist die mediale perimalleoläre Schwellung im Seitenvergleich (Abb. 1a), nach Lohrer und Nauck [20] sogar in 58,6 % der Fälle eine strangförmige Struktur oberhalb des Malleolus medialis tastbar. In 54,2 % sahen sie eine Subluxation oder Luxation der Sehne, wenn der Fuß aus supinierter Position heraus aktiv proniert wurde. Wird dazu *anamnestisch *ein plötzlicher Schmerz beim Sport angegeben, besonders ein Umknicken im Sinne der Pronation-Eversion, meist kombiniert mit Dorsalextension des Fußes oder sogar ein mit Schmerz verbundenes Schnappphänomen, was Lohrer und Nauck in 35,6 % registrierten, kann die klinische Diagnose als gesichert gelten.

Zur *Bildgebung *sind Röntgenbilder des OSG in 2 Ebenen, die dynamische Sonographie (Abb. 1b) und in unklaren Fällen das MRT empfohlen [4]. Da die Luxation der M.-tibialis-posterior-Sehne als Begleitläsion bei Pilon-tibiale-Brüchen, Pronations-Eversions-/Weber-C-/AO-44-C3-Frakturen und auch bei peritalaren Luxationen [33, 40, 42, 43, 49] bekannt ist, wird ein zusätzliches CT erforderlich. Nach Lohrer und Nauck [20] zeigte bei isolierter Luxation der MTPS das Röntgenbild in 14,7 % eine ossäre Avulsion des Retinaculums und sicherte somit die Diagnose, die Sonographie in 66,7 %, das MRT in 75,0 %.

**Abb. 2:**
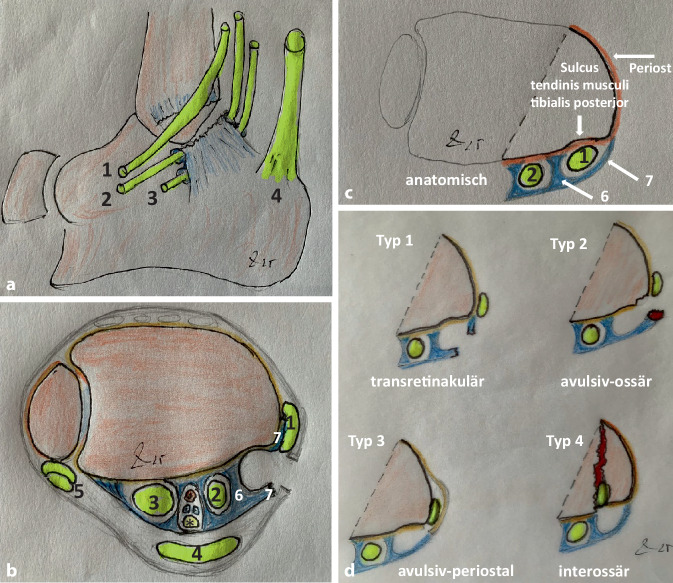
Luxationssituation schematisch dargestellt. **a** Von medial seitlich gesehen: *1* luxierte M.-tibialis-posterior-Sehne, *2* M.-flexor-digitorum-longus-Sehne, *3* M.-hallucis-longus-Sehne, *4* Achillessehne **b** koronare Ebene intraoperativ *1–4* wie in **a**, *5* Mm. peronaei, *6* Stratum profundum retinaculi mm. flexorum, *7* Stratum superficiale retinaculi mm. flexorum, *Asteriskus* tibiales Nerven-Gefäß-Bündel. **c** Situs mit intakten osteofibrösen Köchern. *1* M.-tibialis-posterior-Sehne, undisloziert in situ, *2* M.-flexor-digitorum-longus-Sehne, *6* Stratum profundum retinaculi mm. flexorum, *7* intaktes Stratum superficiale retinaculi mm. flexorum. **d** Pathogenetische Klassifikation der M.-tibialis-posterior-Sehnen-Luxation

## Luxationstypen

Strydom et al. [39] schlugen 2017 eine 3-stufige Klassifikation vor. Danach entspricht der Typ 1a einem ausgerissenen Retinaculum, der Typ 1b einem abgerissenen Retinaculum mit knöcherner Schale, der Typ 2 einem rupturierten Retinaculum und der Typ 3 einem insuffizienten Retinaculum.

Sakakibara et al. [36] unterschieden 2018 sowie 2021 Gkoudina et al. [9] zwei prinzipielle Luxationstypen. Beim Typ 1 rupturiert bei forcierter Pronation-Abduktion oder extremer Dorsalflexion des Fußes das Retinaculum und begünstigt somit das Herausluxieren der Sehne aus dem Sulcus. Beim Typ 2 wird das Flexoren-Retinaculum samt Periost von der Tibia abgelöst, sodass die Sehne in diese große Retinaculum-Periost-Tasche luxiert.

Da in den bisherigen Klassifikationen eine prinzipielle Luxationsvariante fehlt, nämlich die interossäre Luxation der Sehne zwischen zentrales Tibia- und Malleolus-medialis-Fragment bei OSG- oder Pilon-tibiale-Frakturen bzw. bei der Luxatio sub talo lateralis mit Inkarzeration der Sehne zwischen Talus und Kalkaneus, schlägt der Autor eine *pathogenetische Klassifikation* mit 4 prinzipiellen Luxationstypen vor. Danach luxiert die Sehne beim *Typ 1: transretinakulär *durch Zerreißung des osteofibrösen Köchers. Beim *Typ 2: avulsiv-ossär *kommt es zur knöchernen Avulsion des osteofibrösen Köchers. Beim *Typ 3: avulsiv-periostal *wird das Periost am medialen Ansatz des osteofibrösen Köchers abgelöst, wodurch eine große Luxationstasche entsteht. Beim *Typ 4 interossär *wird durch Fraktur oder subtalare Luxation die Sehne bei meist erhaltenem osteofibrösen Köcher interossär inkarzeriert (Abb. 2d).

## Therapie

Bei akuter Luxation aufgrund der Ruptur des osteofibrösen Köchers und palpabler, ausreichender Tiefe des Sulcus, kommt nach Reposition der Sehne die einfache Naht des Retinaculums zur Anwendung [3, 18, 21, 25, 28, 35]. Bei Avulsion des Retinaculums zusammen mit Periost und/oder knöchernem Fragment werden die anatomische Reposition mit transossärer Naht, mit Miniankernaht, Minischraubenfixation, auch die Fibrinklebung und Fixation mit resorbierbaren Stiften, analog osteochondraler Frakturen empfohlen [13, 30, 45, 49]. In Fällen veralteter oder chronischer Luxation mit Fehlen des Sehnenköchers können eine Periostlappenplastik [20, 38], eine sichere Sehnenführung mittels medial distal-gestieltem Achillessehnenspan [2, 35] bis hin zur Sehnensicherung mittels autologem „bone block“ [19] oder Miniplättchen [15] notwendig werden. Bei zu oberflächlichem Sulcus wird von vielen Autoren eine zusätzliche Vertiefung desselben empfohlen [10, 11, 32, 38], bei ausgerissenem Retinaculum-Periost-Komplex kombiniert mit Knochenankernähten [9].

## Fallvorstellung

### Anamnese*.*

Die seinerzeit 23-jährige Balletttänzerin berichtete, dass sie wegen erheblicher Probleme im Bereich des dorsalen Malleolus medialis links beim Zehenspitzentanz zuerst eine lokale Kortikosteroidinjektion erhalten hatte. Wegen Beschwerdepersistenz erfolgte eine operative Revision der Sehne, was einen Monat später eine radikale Revisionsoperation bei putrider Sekretion aufgrund einer Infektion durch *Klebsiella pneumoniae* erforderte. Acht Monate hiernach wurde die Patientin erstmals vorstellig und klagte darüber, dass sie beim Tanz en pointe im medialseitigen Knöchelbereich links Schmerzen habe und ohne gezieltes, straffes Taping der Knöchelregion gar nicht tanzen könne.

### Befund*.*

Bei *klinischer *Betrachtung war der Bereich oberhalb und unterhalb des Innenknöchels links gegenüber rechts deutlich verstrichen (Abb. 1a). Bei aktiver Pronation des Fußes aus der Supinationsposition heraus konnte ein Springen der M.-tibialis-posterior-Sehne ausgelöst werden, was die folgende *dynamische Sonographie *(Abb. 1b) bestätigte. Erst der intraoperative Befund (Abb. 2 und 3) ließ die genaue Pathologie durch einen veralteten, transretinaculären, pathogenetischen Luxationstyp 1 erkennen.

**Abb. 3:**
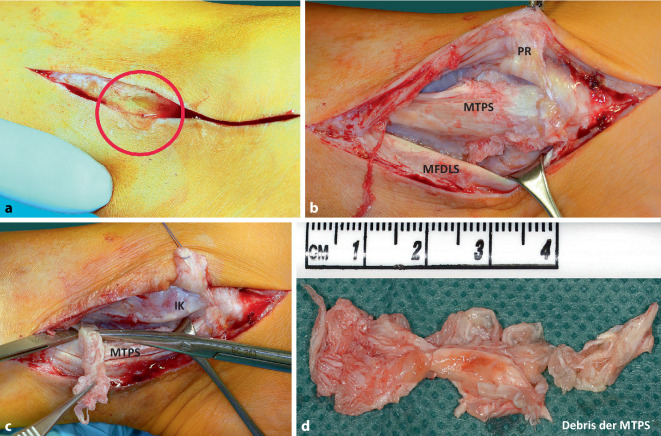
**a** Inzision der medialen Narbe mit Austritt synovialer Flüssigkeit (*roter Kreis*); **b** breit ausgewalzte, nach ventral über den Innenknöchel luxierte M.-tibialis-posterior-Sehne (*MTPS*), die mit einem Pseudo-Retinaculum (*PR*) vernarbt ist, bereits abpräpariert (Typ 1 transretinakulär). Die M.-flexor-digitorum-longus-Sehne (*MFDLS*) wird zum Débridement der MTPS weggehalten: **c,d** 6 cm Débridement der teilrupturierten, fledderigen MTPS. *IK* Innenknöchel

### Indikation*.*

Angesichts der beruflichen Anforderungen bestand eine klare Indikation zur Operation unter gezieltem, perioperativen Antibiotikaschutz.

### Operation*.*

**Abb. 4:**
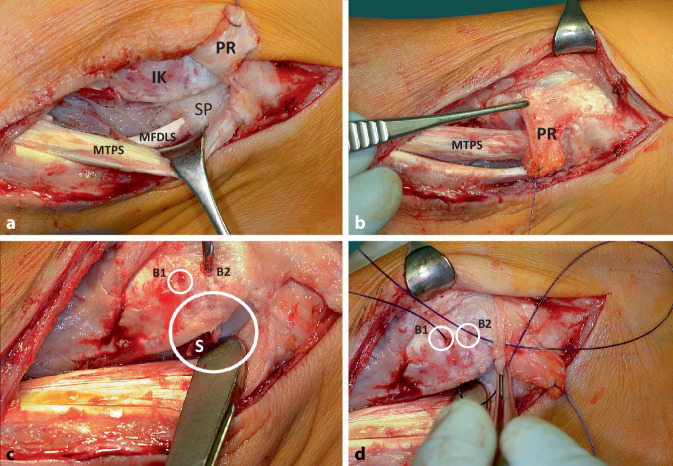
**a** Nach Débridement ist das Stratum profundum retinaculi mm. flexorum (*SP*) erkennbar; **b** PR mobilisiert und rotiert; **c** zwei 2‑mm-Bohrkanäle (*B1* *+* *B2*) werden zum Sulcus tendinis musculi tibialis posterioris (*S*), nahe zur Umschlagskante gesetzt; **d** 2.0 PDS-Fäden ziehen das rotierte PR umschlagskantennah heran

**Abb. 5:**
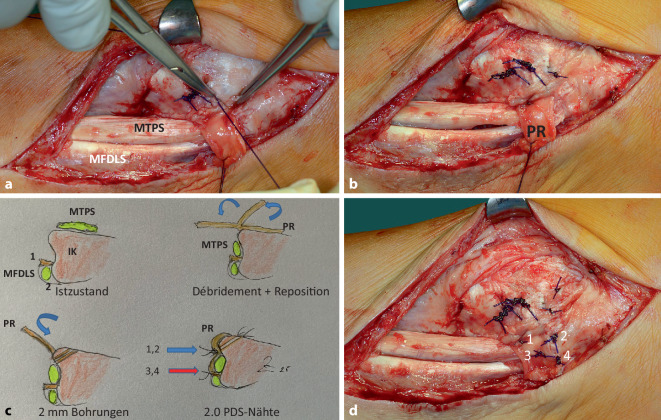
**a,b** Mit 2.0 PDS-Fäden ist das Pseudo-Retinaculum (PR) jetzt umschlagskantennah zum Sulcus tendinis musculi tibialis posterioris hin abgesteppt; **c** Schema zur Rekonstruktion eines Neo-Retinaculums: *MTPS* M.-tibialis-posterior-Sehne, *MFDLS* M.-flexor-digitorum-longus-Sehne, *IK* Innenknöchel, *1* Reststummel des Stratum profundum retinaculi mm. flexorum, *2* erhaltenes Stratum superficiale retinaculi mm. flexorum; *blauer gewundener Pfeil* Hebung und Rotation des PR, *gerader blauer Pfeil* Steppnähte (*1,2*) fürs PR, *roter Pfeil* Nähte des PR ans Stratum profundum i. S. der Köcherrekonstruktion **d** Nähte *3,4* ans Stratum profundum

Vor Anlegen der Blutsperre wurden Antibiotika i.v. entsprechend dem auswärtigen Antibiogramm appliziert. Nach Inzision der medialen Narbe trat reichlich klare synoviale Flüssigkeit aus (Abb. 3a), von der eine Probe zur bakteriologischen Untersuchung genommen wurde. Nach Längsspaltung eines weiten Synovialschlauchs zeigte sich eine über den Sulcusrand (Abb. 3b) nach ventral luxierte, breit ausgewalzte M.-tibialis-posterior-Sehne, die deutlich mit Resten einer retinakulumartigen Struktur (Pseudo-Retinaculum) vernarbt war und von dieser abpräpariert werden musste (Abb. 2b und 3b). Die Sehne erschien über eine Strecke von etwa 6 cm aufgrund zahlreicher Mikro‑/Partialrupturen zerfleddert-ausgefranst und musste sorgfältig, ohne die Sehne zu sehr zu verdünnen, débridiert werden (Abb. 3b–d). Wegen der vorausgegangenen Infektion erfolgten intermittierende Spülungen mit Lavanid® (Polyhexanid). Erst nach dem Débridement der Sehne wurden Reste des Stratum profundum des Flexoren-Retinaculums erkennbar (Abb. 4a). Das präparierte Pseudo-Retinaculum von ca. 1,5 × 4 cm Größe wurde mobilisiert und um annähernd 90° in situ rotiert (Abb. 4b). Nahe zur Umschlagskante des Sulcus tendinis musculi tibialis posterioris wurden zwei 2‑mm-Bohrkanäle unter Schutz der Sehne gesetzt (Abb. 4c). 2.0 PDS-Fäden, transossär geführt, konnten bei Anspannung das rotierte Ersatz-Retinaculum nahe zur Umschlagskannte des Sulcus heranziehen (Abb. 4d und 5a–c). Die Abb. 5a–d zeigt die Führung der Sehne im Sulcus nach Knüpfung der transossären Naht sowie des Ersatz-Retinaculums zum Stumpf des Stratum profundum des Flexoren-Retinaculums (Abb. 5c, d).

### Nachbehandlung*.*

Nach Primärheilung der Wunde erfolgten die Mobilisation im Unterschenkel-Geh-Cast mit 20 kp Teilbelastung für insgesamt 6 Wochen, eine Thromboseprophylaxe sowie die oralen Gaben von Tavanic 500® (Levofloxacin, 2-mal/Tag) und Metronidazol®400 (Nitroimidazol, 3-mal/Tag) bis zum Ende der 3. postoperativen Woche. Ab der 7. Woche nach der Operation folgte eine intensive ambulante Rehabilitation mit Muskelaufbau, Propriozeptiv- und Koordinationstraining. Die Arbeitsfähigkeit war bereits nach 8 Wochen gegeben.

## Ergebnis

Die intraoperativ genommene bakteriologische Probe war negativ. Nach wiedergegebener Arbeitsfähigkeit konnte die junge Balletttänzerin rasch in vollem Umfang schmerzfrei tanzen, benötigte keinerlei Taping mehr und qualifizierte sich an ihrem Opernhaus nach 3 Monaten zur Solistin. Nach heute 13-jährigem Verlauf ist die jetzt 36-jährige Primaballerina nach wie vor auch beim En-pointe-Tanz völlig schmerzfrei. Sie selbst schätzt ihr Tanzvermögen heute höher ein als vor 13 Jahren.

## Diskussion

*Epidemiologisch und pathogenetisch *ist die Luxation der M.-tibialis-posterior-Sehne als Entität seit den 1980er-Jahren mittels Sonographie oder MRT sicher erkennbar. Meist als indirektes Pronation-Eversion-Trauma beim Sport sind auch direkte Verletzungen [15, 26] ursächlich sowie spontane Luxationen ohne Trauma in 15–28 % der Fälle bekannt [1, 20]. Wenngleich seit 2017 zwei MTPS-Luxation-Klassifikationen vorliegen [9, 36, 39], konnten bis heute sowohl anatomische Studien [38] als auch neuere CT-Analysen [46] weder eine Relation zu diesen, noch eine Prädisposition zur Luxation sicher erkennen lassen.

*Pharmakologisch *greift eine lokale Kortikosteroidapplikation bei schmerzhafter Peritendinitis nicht ursächlich an, sondern führt zu einer „Mesenchymnarkose“ [16]. Durch den schwindenden Schmerz werden Training, Wettbewerb und Performance fortgeführt, sodass es zur pathologischen Ruptur des Retinaculums mit Luxation der Sehne oder sogar zur Ruptur derselben [6] kommen kann, wie dies analog bei paratendinöser Kortisoninjektion an der Achillessehne häufig beobachtet wurde und deshalb vor dieser Applikation gewarnt wurde [5, 6, 8, 16, 49]. Nach Untersuchungen an Athleten 2005 von Nichols [29] können Kortikosteroidinjektionen, appliziert bei Sportverletzungen, als die häufigste Komplikation Rupturen von Sehnen und Faszien verursachen. Bloch erklärt 2023 dies experimentell [5] mit negativen Effekten auf die Zusammensetzung der Extrazellularmatrix durch eine gestörte Tenozyten- und Fibrozytenfunktion mit Herunterregelung der Kollagen-I-Expression.

Zum Infektionsrisiko von lokal applizierten Kortikosteroiden konnten Handchirurgen bei intraoperativer Injektion eines Kortikosteroids sowohl in einer retrospektiven [22] als auch in einer prospektiven Studie [14] eine signifikant erhöhte Rate (*p* = 0,03) schwerer Infektionen nachweisen, weswegen diese Autoren bei einem relativen Risiko von 1–33 % vor dieser Anwendung warnen. In einer multivariaten Analyse [7] konnte nachgewiesen werden, dass eine postoperative Infektion nach Karpaltunnelspaltung mit *p* = 0,034 signifikant erhöht war, wenn eine Kortikosteroidinjektion bis 30 Tage präoperativ appliziert worden war.

Ob in dem von Ouzounian und Myerson [31] beschrieben Fall die wiederholten Kortikosteroidinjektionen die MTPS-Luxation induzierten oder die einmalige Injektion im vorliegenden Fall die spätere putride Infektion und Luxation verursachte oder nicht, muss offen bleiben. Auch im kürzlich beschriebenen Fall einer pathologischen Ruptur des distalen peronäalen Retinaculums mit Luxation der M.-peronaeus-longus-Sehne nach Kortisoninjektion bei einem Hochsprungolympioniken blieb diese Frage offen [50].

*Therapeutisch *wird heute allgemein die Operation empfohlen, wenngleich Lohrer und Nauck [20] in deren Review von 61 Fällen bei 10 konservativ behandelten Patienten 3 exzellente bis zufriedenstellende Ergebnisse fanden. Auch der 1874 erstmals beschriebene Fall einer MTPS-Luxation, die der Autor Prof. Charles Martins (1806–1889) selbst bei einer notfallmäßigen Ballonlandung erlitten hatte, war so erfolgreich, dass er bereits nach 3 Monaten schmerzfrei und ohne Stock gehen konnte [23]. Allerdings war diese Luxation durch den ihn begleitenden Chirurgen Dr. Samuel Pozzi (1846–1918) sofort erkannt, die Sehne reponiert, mit einer Notfallbandage stabilisiert und mit 6‑wöchiger Immobilisation im „appareil silicaté“, einem Vorläufer des Gipsverbandes, behandelt worden. Deshalb ist denkbar, dass ein solch gutes Ergebnis auch heute, bei sofortiger Erkennung einer MTPS-Luxation, manueller Reposition der luxierten Sehne und Immobilisation für 6 Wochen gut verheilen kann. Jedoch sind die operativen Ergebnisse sehr gut, gut und ohne Rezidiv, weswegen Perlmann et al. [32] vor unsicherer konservativer Behandlung warnen.

Nach Lohrer und Nauck [20] war in 49 analysierten operativen Fällen in 32,7 % eine direkte Naht möglich, in 42,9 % eine Retinaculum-Rekonstruktion und in 18,4 % eine zusätzliche Vertiefung des Sulcus notwendig. Bei Sonographie- oder MRT-Frühdiagnostik dürfte der Anteil einer direkten Retinaculum-Naht heute höher sein. Durch die Reduktion übersehener, veralteter Fälle, sollten daher aufwendige Operationen wie Tenoskopie mit „suture taping“ [24], eine 2 × 2-cm-Beckenkammspantransposition mit 4,0-mm-Schraubenosteosynthese [19], eine aufklappende Innenknöchelosteotomie mit Miniplättchenosteosynthese [15], Techniken wie Sulcus-Korrektur mit additiver Plättchenosteosynthese [27] oder die retromalleoläre, ampliskopisch-gesicherte Bohrung mit einem Kreuzband-Reamer [47] vermeidbar werden.

## Fazit für die Praxis

Plötzlich auftretende Schmerzen im Innenknöchelbereich beim Sport, insbesondere wenn anamnestisch ein OSG-Trauma im Sinne der Pronation-Eversion angegeben wird, sollten besonders dann differenzialdiagnostisch an eine MTPS-Luxation denken lassen, wenn zusätzlich das Gefühl eines Schnappphänomens geschildert wird. Röntgen des OSG in 2 Ebenen, Sonographie oder MRT sichern die Diagnose. Bei akuter Luxation ist meist die direkte Naht des Retinaculums, die transossäre Retinaculum-Periost-Naht oder die Knochenankernaht zielführend, bei veralteter oder chronischer Luxation die anatomische Rekonstruktion des Retinaculums mit lokalem, körpereigenem Gewebe. Eine zusätzliche Vertiefung des Sulcus ist nur bei unzureichender Tiefe notwendig.
